# THE HANDICAP PROCESS FAVORS EXAGGERATED, RATHER THAN REDUCED, SEXUAL ORNAMENTS

**DOI:** 10.1111/evo.12450

**Published:** 2014-06-12

**Authors:** Samuel J Tazzyman, Yoh Iwasa, Andrew Pomiankowski

**Affiliations:** 1 CoMPLEX, , University College LondonLondon WC1E 6BT, United Kingdom; 2 Institute of Integrative Biology (IBZ)ETH Zürich, Zürich 8092, Switzerland; 3 The Galton Laboratory, , Department of Genetics, Environment, and Evolution, , University College LondonLondon WC1E 6BT, United Kingdom; 4 Department of Biology, Faculty of Science, , Kyushu UniversityFukuoka 812-8581, Japan

**Keywords:** Handicap process, mate choice, mate preference, sexual dimorphism, sexual selection, signaling/courtship

## Abstract

Why are traits that function as secondary sexual ornaments generally exaggerated in size compared to the naturally selected optimum, and not reduced? Because they deviate from the naturally selected optimum, traits that are reduced in size will handicap their bearer, and could thus provide an honest signal of quality to a potential mate. Thus if secondary sexual ornaments evolve via the handicap process, current theory suggests that reduced ornamentation should be as frequent as exaggerated ornamentation, but this is not the case. To try to explain this discrepancy, we analyze a simple model of the handicap process. Our analysis shows that asymmetries in costs of preference or ornament with regard to exaggeration and reduction cannot fully explain the imbalance. Rather, the bias toward exaggeration can be best explained if either the signaling efficacy or the condition dependence of a trait increases with size. Under these circumstances, evolution always leads to more extreme exaggeration than reduction: although the two should occur just as frequently, exaggerated secondary sexual ornaments are likely to be further removed from the naturally selected optimum than reduced ornaments.

The existence of secondary sexual ornaments, and of sexual preferences for them, is a theoretically well-understood phenomenon, with many mathematical models showing how ornament/preference evolution can arise (Mead and Arnold 2004; Kuijper et al. 2012). However, models do not account for one notable phenomenon: that secondary sexual ornaments generally seem to be larger than the naturally selected optimum size, rather than smaller. Existing models of preference/ornament evolution generally treat reduction and exaggeration as symmetrical (Lande 1981; Pomiankowski and Iwasa 1993; van Doorn and Weissing 2006) or allow for just a single direction of trait evolution, which could then be interpreted as being either exaggeration of the trait, or reduction (Kirkpatrick 1982). Thus, we would expect the two possibilities to be equally prevalent.

In some cases, there is no possibility for ornamental traits that are reduced in size, because the naturally selected size is to have no ornament (e.g., the ornamental leg tufts in wolf spiders *Schizocosa crassipes*; Hebets and Uetz 2000). However, in many other cases, mating preference is for exaggerated versions of already-existing morphological traits, and so an ornamental reduction in size (and/or a preference for smaller traits) would be possible (e.g., eye-stalk length in stalk-eyed flies; Wilkinson and Reillo 1994; Cotton et al. 2006). Despite this possibility for reduction, few if any species seem to exhibit reduced ornaments, or preferences for them (for a thorough review, see Tazzyman et al. 2014).

Under the framework of Fisher 's runaway process (Fisher 1999), the preponderance of exaggeration rather than reduction can best be explained by the fact that the signaling efficacy of an ornament increases with size (Tazzyman et al. 2014). The loss of signaling efficacy in smaller traits means that runaway is prevented in the direction of reduction, whereas it is still possible in the direction of exaggeration. However, the runaway process must in reality halt somewhere, and Fisher 's process is only one potential explanation for the evolution of secondary sexual ornamentation. Another well-studied possibility is the handicap process (Zahavi 1975). To fully explain the reasons why trait exaggeration is apparently so much more common than trait reduction, and hence to address this gap in the current theory of the evolution of sexual signaling, we need to understand the extent to which the handicap process also contributes to sexual trait size asymmetry.

Under the handicap process, high-quality individuals must have higher marginal fitness benefits from advertising (Grafen 1990; Iwasa et al. 1991; Getty 1998; Holman 2012), either because for them the cost of ornament expression increases more slowly (condition-dependent handicap), or because the benefit in terms of mating success increases more rapidly (revealing handicap) (van Doorn and Weissing 2006). We focus on condition-dependent handicaps, and consider the costs of such traits. It is not only the case that ornamental traits larger than the naturally selected optimum impose a cost on their bearer; traits smaller than the naturally selected optimum are also maladaptive, and could thus be handicap traits. A priori, the handicap principle should be just as likely to lead to reduced ornaments as to exaggerated ornaments, something born out in theoretical models (either implicitly, e.g., Pomiankowski 1987; Iwasa et al. 1991; Iwasa and Pomiankowski 1994; Kirkpatrick 1996; or explicitly, e.g., Iwasa and Pomiankowski 1995; Pomiankowski and Iwasa 1998; van Doorn and Weissing 2006). Some additional factors must therefore explain the disparity between exaggeration and reduction. Here we aim to investigate what these factors might be.

We consider four potential explanations: three following a previous study of Fisher 's process (Tazzyman et al. 2014), plus an additional one only applicable to handicap traits. First, it is likely that as trait size increases so does signaling efficacy, because, for example, larger traits will likely be visible to potential mates from further away (Endler 1993; Leichty and Grier 2006; Fawcett et al. 2007), and will be easier to compare. As mentioned above, incorporating an increased signaling efficacy with increased ornament size was previously found to be a viable explanation for the preponderance of exaggerated traits in cases where secondary sexual ornaments and mate choice preferences evolve by Fisher 's runaway alone (Tazzyman et al. 2014), so it seems reasonable to investigate whether this also applies to the handicap process.

A second potential explanation is that mate choice preference is unequal when it comes to exaggerated and reduced ornaments. As mentioned above, exaggerated ornaments are likely to be easier to see, and consequently preference for them may impose a smaller cost than preference for reduced ornaments. Cost of preference can be assumed to increase as the preference becomes stronger, but perhaps the rate of this increase is greater in the case of preferences for reduced ornamentation than in the case of preferences for exaggerated ornamentation. If this is true, perhaps the evolution of preference for exaggerated ornaments is favored over the evolution of preference for reduced ornaments, because choosy mates are able to accrue the same benefits (high-quality mates) for a smaller cost.

A third possibility is that the costs of ornaments themselves are different depending upon whether the ornament is exaggerated or reduced. The element of cost for ornamental traits is crucial to the handicap principle, and these costs will naturally increase as an ornament deviates in size from the naturally selected optimum. However, the rate of this increase may differ depending on the direction of deviation. If this rate of increase in cost is greater for reduced ornaments than for exaggerated ornaments, it may be that the evolution of exaggerated ornaments is favored, as the balance point at which cost of ornament equals benefit of increased number of matings may be more extreme in the exaggerated case.

Finally, the fourth possibility is that the degree to which an ornament is condition dependent differs depending on whether the ornament is exaggerated or reduced. Exaggerated ornaments will require more resources for their production than reduced ornaments, because they are by definition larger. Although both types of ornament might be equally costly in terms of being not the optimal size, the ability to accrue the required resources to construct the ornament will also affect fitness. Because higher viability males are likely to be able to accrue resources more easily than lower viability individuals, condition dependence is likely to be more pronounced in the exaggerated direction than in the reduced direction.

In this study, we build on a classic model of the evolution of secondary sexual ornaments and preferences by the handicap process (Iwasa et al. 1991; Iwasa and Pomiankowski 1994), and incorporate each of these four potential explanations in turn to investigate whether they are able to theoretically explain the imbalance between exaggeration and reduction seen in the real world.

## The Model

### THE BASIC PROBLEM

We follow a well-established quantitative genetic model of handicap evolution. For full details of the model 's background, we refer the interested reader to Iwasa et al. (1991) and Iwasa and Pomiankowski (1994). The sex that bears the sexual ornament is called “male,” and the sex that exerts mate preference is “female,” although the model would equally apply the other way around. Males have two traits, ornament size *s* (measured on a logarithmic scale) and viability *v*. Females have two traits, viability *v* and mating preference *p*.

The log fitness functions for males and females are, respectively,

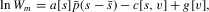
1

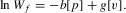
2The first part on the right-hand side of (1) is a male 's fitness due to sexual selection. If mean female preference is 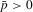 then males with *s*-values greater than the mean 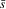 benefit, whereas if 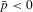 males with *s*-values less than the mean 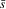 benefit. The amount of benefit is dependent upon the efficacy function 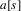, which must be of a form 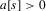 for all *s*. The null situation is to have 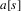 as a constant. We investigate what happens when efficacy increases with ornament size, that is, where 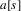 is an increasing function of *s*.

The second part on the right-hand side of (1) is the cost of bearing an ornament of size *s* given that the male is of viability *v*. Costs must always be non-negative, so 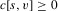 for all *s*, *v*. We define the naturally selected optimum ornament size to be 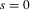. The cost of an ornament then increases the further its size is from 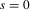. Thus for 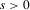, the partial derivative 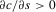 (costs increase as *s* moves away from 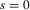), whereas for 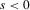, 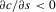 (again costs increase as *s* moves away from 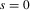). In a handicap model, the rate of increase of cost as ornament size deviates from 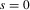 is smaller for more viable males, so the partial derivative

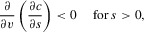


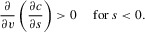


Previous models have assumed that the function 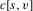 is symmetric, so that for a given *s*, *v*, 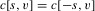. We investigate the evolutionary consequences when exaggerated ornaments are less costly than reduced ornaments, that is, where 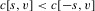.

The final part on the right-hand side of (1) is the boost to fitness of having a higher viability *v*. The function 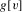 is a monotonically increasing function of *v*.

Equation (2) considers female fitness. The first part on the right-hand side of (2) is the cost of having a preference. Costs must always be non-negative, so 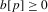. We take 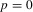 to be the naturally selected optimum, which corresponds to no mating preference. Values of *p* greater than zero correspond to a preference for males with ornaments larger than the population mean, whereas values of *p* less than zero correspond to a preference for males with ornaments smaller than the population mean. The greater the magnitude 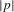, the stronger the preference, and the more costly. So for 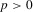, 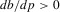 (costs increase as *p* moves away from 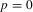), whereas for 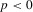, 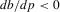 (again costs increase as *p* moves away from zero). Previous models have assumed that the function 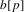 is symmetric, so that 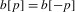 for all *p*. We investigate what happens if preference for exaggerated ornaments is less costly than preference for reduced ornaments, that is, 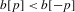.

### DYNAMICS

We suppose that the ornament size *s* of a male is determined by a trait *t*, which represents the condition-independent element of ornament size, his viability *v*, and a further trait 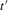, which corresponds to the condition dependence of the ornament, so that 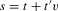. We then track the evolution of the traits *t*, 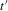, and *p*. The dynamics of the mean traits 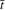, 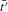, 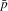, and 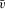 are given by

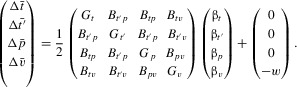
3The factor of 1/2 indicates the sex-limited expression of the traits. The matrix **G** has entries 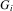 and 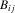, which are, respectively, the additive genetic variance of *i*, and the additive covariance of *i* and *j*. The selection gradients 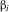 are given by

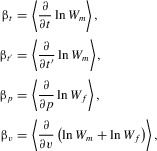
4where 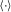 denotes the population mean. The final term on the right-hand side of (5) is the mutation pressure on viability. It is supposed that mutation on viability is biased and so reduces *v*.

We follow Pomiankowski and Iwasa (1993) in decomposing the model into fast and slow dynamics. Because the mutation bias *w* and the rate of increase of cost of female choice 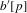 are both likely to be small, the system will first evolve to the neighborhood of the quasi-equilibrium line, along which 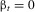, 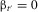, and 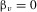. After this the slow dynamics will take over and the system will evolve along the quasi-equilibrium line.

### FAST DYNAMICS

Along the quasi-equilibrium line, 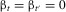. From (1) and (6) this gives

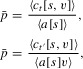
5where 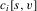 denotes the derivative of 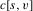 with respect to *i* evaluated at 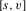. Equating the two equations of (7) gives the relations between 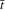, 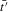, and 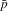 after the conclusion of the fast dynamics. By making specific assumptions about the functions 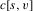 and 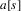, we can find explicit solutions to these equations.

### SLOW DYNAMICS

Once the system has converged to the neighborhood of the quasi-equilibrium line, its behavior is governed by the slow dynamics. We follow other population genetics results (Barton and Turelli 1991; Pomiankowski and Iwasa 1993; Iwasa and Pomiankowski 1994; Tazzyman et al. 2014) in calculating that along the quasi-equilibrium line, to leading order,

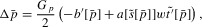
6where 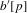 is the derivative of the cost function 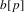 evaluated at *p*, 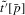 is the quasi-equilibrium value of 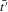 given 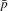, calculated from (7), and 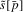 is the quasi-equilibrium value of mean ornament size 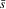 given 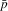 (see Appendix for full details). From (8) we can calculate the equilibrium values 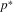 for which 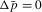.

### LARGER ORNAMENTS ARE BETTER SIGNALS

Previous quantitative genetics models of sexual selection have used a “psychophysical” approach to signaling efficacy, based on Weber 's law, so that the ability to discern differences between two traits is proportional to the relative sizes of the two traits (Stevens 1975; Lande 1981). Because ornament size is measured on a log scale, this corresponds in our model to a constant value of *a*. We want to change the basic model so that 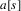 is instead an increasing function of *s*: the relative size differences between ornaments are easier to discern for larger ornaments. Because we wish to leave the other components of the model unchanged, for 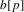 and 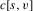 we follow Iwasa and Pomiankowski (1994), so that

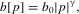


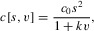


where 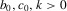, and 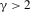. This means that as preference *p* deviates from the 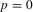 “no preference” case, the cost of preference increases as a power function, with exponent γ and coefficient 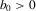. This is true whether preference is for ornaments larger than the mean (i.e., 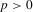) or smaller (i.e., 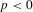). As ornament size *s* deviates from the 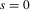 “no ornament” case, cost goes up as a quadratic, with coefficient 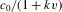, 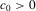, 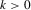, reflecting the fact that males with higher viability *v* will pay lower costs for the same ornament. The parameter *k* thus represents the condition dependence of the cost of the ornament.

### PREFERENCE FOR SMALLER ORNAMENTS COSTS MORE

We take 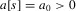, a constant, as in previous models of sexual selection, and as above take

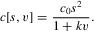


We now alter 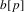 so it is less costly for females to prefer larger ornaments than to prefer smaller ornaments:

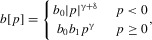


for constants 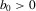, 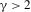, 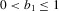, and 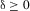. Thus, as preference deviates from the 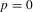 “no preference” case in either direction, the cost of preference increases as a power function. However, in the positive direction, the power exponent is γ, whereas in the negative direction it is 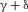. In addition, the coefficient is 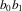 in the positive direction, and *b*_0_ in the negative direction. Thus for 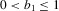, and 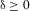, costs of preference increase more rapidly in the negative direction than they do in the positive direction (as long as we do not have both 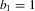 and 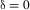).

### SMALLER ORNAMENTS COST MORE

We set 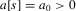 and 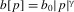, where 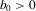 and 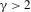. Then we define

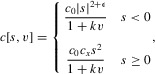


with 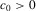, 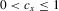, 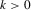, 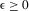. The costs of bearing an ornament increase as a power function as ornament size deviates from 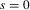. However, in the positive direction the power exponent is 2, whereas in the negative direction it is 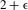. The coefficient in the positive direction is 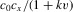, whereas in the negative direction it is 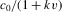. Thus for 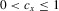, and 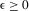, ornamental costs increase more rapidly in the negative direction than they do in the positive direction (assuming we do not have both 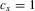 and 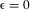).

### LARGER ORNAMENTS ARE MORE CONDITION DEPENDENT

We keep all elements of the model unchanged from the standard form, but we replace the coefficient of condition dependence *k* with a function 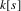 of ornament size *s*. We then have 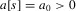, 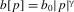, and

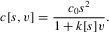
7The function 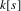 could take many different forms, but for mathematical tractability (see Appendix) we here take 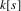 to be simply



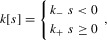
8where 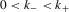 so that condition dependence is greater in the exaggerated direction than in the reduced direction.

## Results

Along the quasi-equilibrium line given by the fast dynamics (7), the relationship between 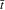 and 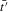 evolves to be fixed, so at any point along this line we can describe 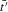 as a monotonically increasing function of 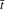. To investigate the evolution of ornament size 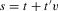, it is then sufficient to consider the evolutionary behavior of 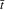 and 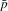 along the quasi-equilibrium line. The larger the magnitude of an equilibrium value of 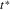 (or 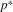), the further from the naturally selected optimum the ornament (or preference) will evolve to be.

### LARGER ORNAMENTS ARE BETTER SIGNALS

**Figure 1:**
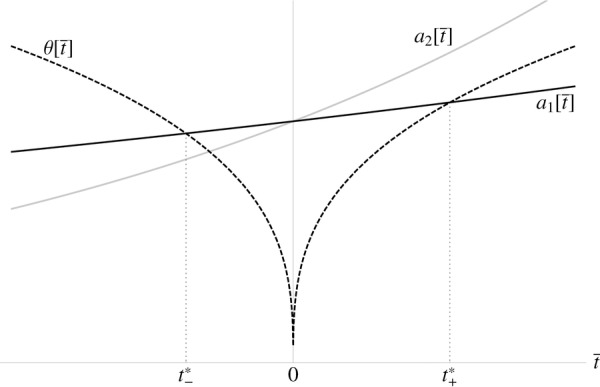
Two examples where larger ornaments are better signals. The system evolves to the quasi-equilibrium line, along which 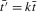. Then the average ornament size 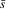 can be expressed as a function of 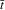, and so we need to only consider equilibrium values of 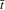. These equilibrium values occur where 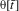 (dashed heavy black line) is equal to 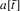 (eq. 10). The solid black line, denoted 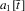, represents a case where there is a gradual increase in signaling efficacy as ornament size increases. There are two values for which 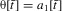, marked 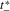 and 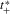. 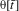 is symmetrical about 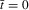, and 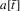 is increasing in 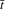, so 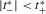. The gray line, denoted 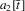, represents the case where efficacy increases more rapidly as ornament size increases. In this case there is a reduced equilibrium where 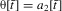, and the gray line meets the dashed black line, but signaling efficacy increases so rapidly in the positive direction that the two curves do not meet, and the system will runaway in the direction of exaggeration. In all cases, evolution toward exaggeration will lead to more extreme ornaments than evolution toward reduction. For this example we have 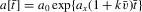, and parameter values 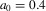, 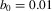, 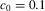, 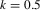, 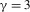, 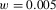, 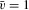, 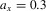 (solid black line), and 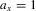 (gray line).

The quasi-equilibrium line (7) after the fast dynamics phase is over is given by

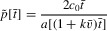
9(eq. (A4) in the Appendix). Then for the slow dynamics, (8) becomes

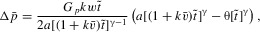
10(eq. (A6) in the Appendix), where

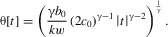
11The equilibria are therefore 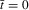 (so that 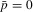) and any nonzero points 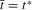 for which 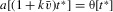 (eq. 10). There will be exactly one negative equilibrium point 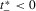, which will be stable. There may be no positive equilibria, in which case the system will run away in a positive direction. Alternatively there may be one or more positive equilibria, in which case the smallest of them will be stable, denoted 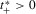. If this stable positive equilibrium exists, then because 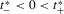 and 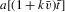 is increasing in 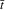, we have 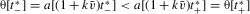. Then by the definition of 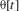 we know that 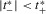 wherever 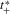 exists (Fig. 1): the exaggerated equilibrium will be more extreme than the reduced equilibrium.

Our results show that if the signaling efficacy of an ornamental trait increases with size, one of two things can occur. First, there can be both an exaggerated and a reduced equilibrium, but the exaggerated equilibrium is further from the naturally selected optimum, so that exaggerated traits grow to be more extreme than reduced traits (seen in Fig. 1 for the solid black efficacy function 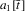). Second, there can be only a reduced equilibrium, but no exaggerated equilibrium, so that reduced traits grow to some fixed size, but exaggerated traits continue to increase in size with every generation in a runaway (seen in Fig. 1 for the gray efficacy function 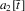). Either way, if larger ornaments are better signals than smaller ornaments, evolution is always biased toward trait exaggeration.

### PREFERENCE FOR SMALLER ORNAMENTS COSTS MORE

The fast dynamics proceed exactly as in Iwasa and Pomiankowski (1994), so that we have 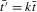 and 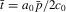 along the quasi-equilibrium line. The slow dynamics phase (8) is then

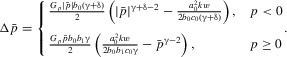
12The equilibrium points are 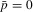, 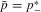, and 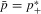, where

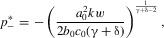
13

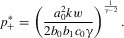
14The equilibrium at the origin is unstable, whereas 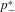 and 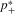 are both stable (Appendix).

**Figure 2:**
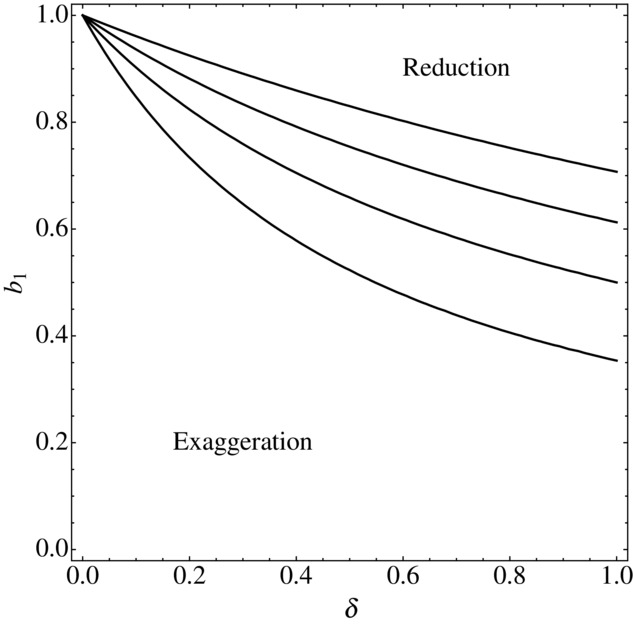
The case where preference for reduced ornaments costs more can lead to both exaggeration and to reduction, depending on parameter values. The curves show the value 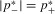, from equations (20) and (21), for different combinations of *b*_1_ and δ. The four curves show the cases where the combined parameters 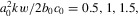 and 2, from the lowest to the highest. The area above each curve is the region in which 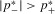, so that the magnitude of the reduced preference is greater than that of the exaggerated preference. The area below each curve is the region in which 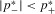, so that the magnitude of the reduced preference is less than that of the exaggerated preference. The other parameter 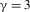.

Because the equilibrium values are 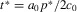, the sizes of the positive and negative equilibrium ornament sizes will depend on the sizes of the positive and negative equilibrium preferences. If 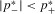, then the exaggerated equilibrium ornament size will be more extreme than the reduced equilibrium ornament size. This is the case if 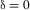 and 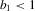. More generally, however, we cannot be sure which of the two equilibria has the greater magnitude: if the term in brackets on the right-hand side of equation (21) has magnitude less than 1, then it is possible that 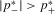, and the reduced equilibrium could be the more extreme of the two (Fig. 2). Thus, it is not true that an increased cost of preference for smaller ornaments *necessarily* results in the exaggerated equilibrium being more extreme than the reduced equilibrium, nor is there a possibility of runaway evolution in either direction.

Our results show that when preference for smaller ornaments is more costly, there will be both an exaggerated and a reduced equilibrium, so that if evolution were to proceed in either direction it will come to rest with some ornamental trait that differs from the naturally selected optimum size, and a related preference. However, we cannot say that exaggerated traits are likely to be more extreme than reduced traits, because the exact details of the two equilibria will depend upon parameter values (Fig. 2).

### SMALLER ORNAMENTS COST MORE

After some calculation (see Appendix for details) we obtain the quasi-equilibrium line

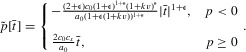


**Figure 3:**
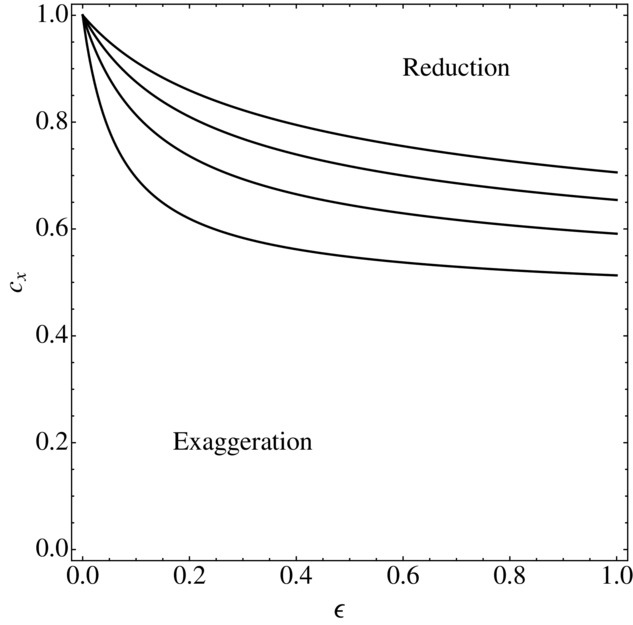
The case where smaller ornaments cost more can lead to both exaggeration and to reduction, depending on parameter values. The curves show the value 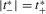, from equations (25) and (26), for different combinations of 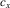 and ε. The four curves show the cases where 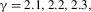 and 2.4, from the lowest to the highest. The area above each curve is the region in which 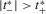, so that the magnitude of the reduced ornament is greater than that of the exaggerated ornament. The area below each curve is the region in which 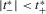, so that the magnitude of the reduced ornament is less than that of the exaggerated ornament. The other parameters 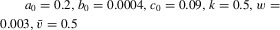.

The slow dynamics (8) are then

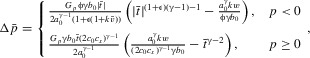
where

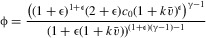
(see Appendix). The equilibrium points are 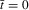, 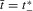, and 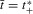, where

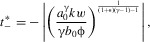
15

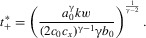
16The origin is unstable, whereas 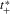 and 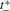 are stable (Appendix). If 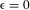 and 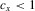, then 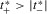, and the exaggerated equilibrium ornament size will be more extreme than the reduced equilibrium ornament size (Appendix). More generally, however, we cannot be sure which of the two equilibria will be the more extreme: if the terms in brackets on the right-hand sides of equations (25) and (26) both have magnitude less than 1, it is possible that 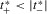, so that the reduced equilibrium is the more extreme of the two (Fig. 3). Thus it is not true that an increase cost of smaller ornaments *necessarily* results in the exaggerated equilibrium being more extreme than the reduced equilibrium, nor is there a possibility of runaway evolution in either direction.

Similar to the case above for costs of preference, our results show that when smaller ornaments are more costly, there will be both an exaggerated and a reduced equilibrium, so that if evolution were to proceed in either direction it will come to rest with some ornamental trait that differs from the naturally selected optimum size, and a related preference. However, we cannot say that exaggerated traits are likely to be more extreme than reduced traits, because the exact details of the two equilibria will depend upon parameter values (Fig. 3).

### LARGER ORNAMENTS ARE MORE CONDITION DEPENDENT

Finally, we can consider the case where smaller ornaments are less condition-dependent than larger ornaments, following equations (14) and (15). After some calculation (see Appendix for details), we get the quasi-equilibrium lines 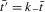 for 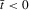, and 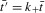 for 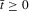, giving 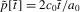 in both cases. The slow dynamics (8) are then

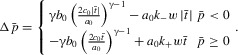
The equilibrium points are then 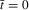, 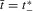, and 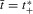, where

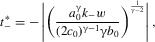


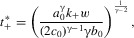
(see Appendix) and because 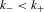 we have 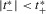. The origin is unstable, whereas 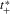 and 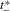 are stable.

Our results show that if condition dependence is greater in the direction of exaggeration than in the direction of reduction, we always have two equilibria: one in the direction of exaggeration, and one in the direction of reduction. The exaggerated equilibrium is further from the naturally selected optimum than the reduced equilibrium, so that exaggerated traits will grow to be more extreme than reduced traits.

## Discussion

Previous models of the evolution of secondary sexual ornaments have presumed a symmetry between exaggerated and reduced traits, whereas in nature this symmetry is notably absent, with exaggerated traits apparently much more frequent than reduced traits (Ryan and Keddy-Hector 1992; Tazzyman et al. 2014). We used a quantitative genetics approach, adapting a classic model (Iwasa et al. 1991; Iwasa and Pomiankowski 1994) to incorporate four different possible explanations for this asymmetry when secondary sexual ornaments evolve via the handicap process (Zahavi 1975).

The first explanation was that signaling efficacy is an increasing function of trait size. This is biologically reasonable, because exaggerated traits are likely to be more easily visible by would-be mates, and may also be more reliable as signals. We showed that adding this signaling efficacy asymmetry to existing models (Iwasa et al. 1991; Iwasa and Pomiankowski 1994) means that exaggeration will produce more extreme ornamentation than reduction (i.e., ornaments further from the naturally selected optimum). The fact that signaling efficacy increases with trait size is potentially sufficient to explain the imbalance between exaggeration and reduction seen in the real world. When secondary sexual ornaments evolve to be reduced in size, they incur increased cost and decreased efficacy as they diverge from the naturally selected optimum. When they evolve to be increased in size, they incur increased cost but also gain increased efficacy as they diverge from the naturally selected optimum. Consequently, the equilibrium ornament size is further removed from the naturally selected optimum in the exaggerated case than in the reduced case. Indeed, if the gain in efficacy is greater than the increased cost as ornaments become larger, runaway occurs in the direction of trait exaggeration. Such a runaway cannot occur in the direction of trait reduction.

The second potential explanation was to do with the cost of female preference. In particular, we investigated the case where the rate of increase of cost as female preference becomes stronger is greater in the reduced direction than in the exaggerated direction: for a given strength of preference, females who prefer reduced males pay a higher cost than females who prefer exaggerated males. Biologically this explanation again seems plausible: assessing reduced ornaments is likely to be harder than assessing exaggerated ornaments. However, support for this explanation was equivocal: whereas for some parameter values this framework results in the equilibrium for exaggerated ornamentation being more extreme than that for reduced ornamentation, this is not necessarily the case for all parameter values. For some parameter combinations the reverse is true, with reduced ornamentation being the more extreme of the two (Fig. 2). In addition, under this framework runaway evolution is impossible in either direction (using the functions we investigated above).

The third potential explanation was to do with the cost of the secondary sexual ornament. We investigated the case where the rate of increase of cost as a trait deviates from the naturally selected optimum is greater in the reduced direction than it is in the exaggerated direction: for a given deviation from the naturally selected optimum, a reduced trait will be more costly than an exaggerated trait. Biologically this is difficult to justify: a priori it seems feasible that the reverse would be true, because all else being equal the resources needed to construct an exaggerated ornament are by definition greater than the resources needed to construct a reduced ornament. However, it could still potentially be true at least in some cases. Again, however, the theoretical support for this possibility provided by our model was equivocal. For some parameter values, the equilibrium point for exaggerated ornamentation is further from the naturally selected optimum than the equilibrium for reduced ornamentation, meaning that exaggerated ornamentation is more extreme than reduced. However, for other parameter values the reverse is true, and reduced ornamentation is the more extreme (Fig. 3). In addition, runaway evolution is impossible in either direction under this framework (using the functions we investigated above).

The final possibility we investigated was that the condition dependence of the ornament, which is crucial for the ornament to be a handicap trait, differed between exaggerated and reduced traits. We supposed that exaggerated traits were more condition dependent than reduced traits. Biologically this is plausible on the grounds that exaggerated traits are likely to require more resources to grow and maintain, and consequently are likely to be harder for low-condition males to attain. Reduced traits, on the other hand, whereas also being costly due to their suboptimal size, require fewer resources to grow, and so may be more attainable for low-condition males. In the simple case where condition dependence is fixed for both exaggerated and reduced traits, and is greater in the former than in the latter, exaggeration will produce more extreme ornaments than reduction. To see why, note that ornaments with greater condition dependence provide females with more information, and consequently females gain greater benefits from preferences for exaggerated traits. Therefore the point at which the cost of preference balances the benefit of mating with a male in good condition will be at a more extreme preference level in the exaggerated case than in the reduced case. This provides a second potential explanation for the preponderance of exaggerated traits in the real world. If ornaments largely evolve through the handicap process, and condition dependence is greater for exaggerated traits than for reduced traits, then exaggerated traits will be more extreme than reduced traits. However, we stress that we have only investigated this case for a simple characterization of condition dependence. Although it is often assumed that the exaggerated size of secondary sexual ornaments implies that they are more condition dependent, in fact this is not certain (Cotton et al. 2006), and the condition dependence of a sexual ornament at equilibrium will depend on the exact form of the cost function (Johnstone et al. 2009). We have shown for a simple cost function that evolution is likely to favor more extreme traits in the direction of increased condition dependence. It would be worthwhile to investigate the case for more complicated cost regimes, and also to establish whether exaggeration is necessarily more condition dependent than reduction.

We have previously shown that where a trait evolves purely through the Fisher process, the most likely explanation for the preponderance of exaggerated traits is that the efficacy of a signaling trait is likely to increase as the trait increases in size (Tazzyman et al. 2014). This result is echoed here for the handicap process, with signaling efficacy again a potential reason for the observed fact that secondary sexual traits are generally more exaggerated than reduced. We have also shown that condition dependence might play a role. Support for signaling efficacy and for condition dependence is strong because these two explanations alone will *always* bias the system in favor of exaggeration and against reduction. Our work here does not rule out the other two explanations (increased costs of preference or trait in the reduced direction), which could also provide the kind of asymmetry required. But in this case, bias toward exaggerated trait values is dependent upon a restricted set of parameter values. Similarly, our previous work on the Fisher process showed that only signaling efficacy necessarily provided the asymmetry required, but an increased cost of trait in the reduced direction could have also provided an explanation for a restricted set of parameter values (Tazzyman et al. 2014). For the handicap process, we are able to suggest that traits will generally evolve in the direction of increased signaling efficacy and/or in the direction of increased information content (i.e., increased condition dependence), but this tendency could be affected by asymmetries in costs of signal or preference.

The explanations here need not be mutually exclusive; it is easy to believe, for example, that exaggerated ornaments are simultaneously more efficacious as signals and less costly for females to prefer. The effects of this are difficult to assess, because the models above are technically difficult to analyze, particularly for the two explanations that feature asymmetrical costs. We suspect that because the results are so simple and clear in the cases of increased signaling efficacy and increased condition dependence, they would likely carry over to more complicated scenarios in which there were multiple asymmetries acting simultaneously (as is probably likely in reality).

Our work can be seen as being relevant to ideas about sensory bias and secondary sexual ornament evolution, because it is possible to conceive of the direction of sensory bias being that in which signal efficacy increases with ornament size. Although we do not show that signaling traits are more likely to evolve toward greater signaling efficacy (Endler et al. 2005), we do show that if they do evolve in this direction, they will likely become more exaggerated.

It is notable that our model still results in symmetry between exaggerated and reduced secondary sexual ornaments in the sense that a system starting at the origin (no preference) is equally likely to evolve in the exaggerated or the reduced direction. The only differences are the magnitude of the equilibrium that the system reaches differs depending on in which direction evolution proceeds, and that runaway evolution can only ever occur in the exaggerated direction. Our work, just like other theoretical models of sexual ornament and preference evolution, does not suggest that exaggerated traits should evolve more frequently than reduced traits (Mead and Arnold 2004; Kuijper et al. 2012; Tazzyman et al. 2014). This leads to the prediction that there are equally as many reduced ornaments as exaggerated ornaments in the real world. In reality, however, very few examples are known of reduced secondary sexual ornaments. Possible examples are the golden-headed cisticola *Cisticola exilis* (Balmford et al. 2000), and the fairy wren *Malurus melanocephalus* (Karubian et al. 2009), but there are problems in both cases, and few other possibilities (Tazzyman et al. 2014). This may be due to reporting bias. Because of the effects of signaling efficacy increasing with ornament size, reduced ornaments might be harder to observe because they are similar in size to the naturally selected optimum. The exaggerated ornaments, on the other hand, are more extreme, and so are noticed. It may be that if reduced ornaments are carefully looked for, they will be found to be as frequent as exaggerated ornaments. On the other hand, there may still be something missing in the theory of ornament and preference evolution.

A possible answer to this lack of reduced ornamental traits could be found by looking at a nonequilibrium dynamical model of ornament-preference evolution. Previous models have shown that in some cases equilibria for trait and preference do not exist, for example, because of the costs of preference (Iwasa and Pomiankowski 1995; Pomiankowski and Iwasa 1998), the exhaustion of good genes variation due to extreme ornamentation (Houle and Kondrashov 2002), or conflict over the information content of ornamental signals (van Doorn and Weissing 2006). In these cases continual evolution occurs, with traits and preferences cycling in complicated ways depending upon parameter values. Some of these models (Iwasa and Pomiankowski 1995; Pomiankowski and Iwasa 1998; van Doorn and Weissing 2006) explicitly feature negative values of preference and ornament, and the resulting evolutionary trajectories cycle through this negative portion of evolutionary space just as frequently as through the positive portion, meaning existing nonequilibrium models also predict that reduced and exaggerated traits (and the preferences for them) should be equally frequent. We suggest that a logical next step in the investigation of the lack of reduced ornamental traits would be to see what effect asymmetries in cost of ornament, cost of preference, condition dependence, and signaling efficacy, have on nonequilibrium models.

In conclusion, we have shown that the preponderance of exaggerated traits seen in nature could be at least partly explained by our finding that secondary sexual ornaments are likely to reach a more extreme equilibrium when they evolve via the handicap process in the direction of increased signaling efficacy (which we have previously shown to also be true for Fisher 's process, Tazzyman et al. 2014), or the direction of increased condition dependence. However, in the case of the handicap process, these explanations also lead to the further prediction that there are in fact just as many reduced traits as exaggerated traits, but they are closer in size to the naturally selected optimum. Therefore, we suggest that further work is needed before it can be concluded that this problem has been fully solved.

## Figures and Tables

**Figure 4:**
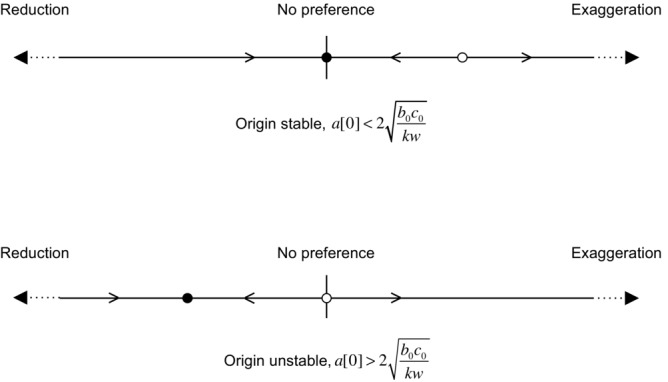
Two possibilities for the system when signaling efficacy is an increasing function of signal size, and 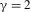. For both, the central point is marked as “no preference” and shows the equilibrium where there is no preference, and the ornamental trait remains at the naturally selected optimum. Ornamental exaggeration is toward the right, ornamental reduction toward the left. The first line shows the situation when the signaling efficacy at the naturally selected optimum is below the threshold given by equation (62). The origin is stable (marked by a filled circle), and secondary sexual signaling does not evolve unless some stochastic fluctuation pushes the system to the right of the unstable equilibrium (marked by the empty circle). The second line shows the situation when the signaling efficacy at the naturally selected optimum is above the threshold given by equation (62). The origin becomes unstable (marked by an empty circle). The system either runs away toward exaggeration (to the right), or evolves to the reduced equilibrium (marked by the filled circle). If the signaling efficacy at the naturally selected optimum equals the threshold then the only equilibrium will be at the origin. It will be stable to perturbations toward ornamental reduction, but will run away toward exaggeration.

## Appendix

### GENERAL SLOW DYNAMICS

The method we follow is taken from Iwasa and Pomiankowski (1994, Appendix). We go through it very briefly here. Following the conclusion of the fast dynamics, , , and  are somewhere near quasi-equilibrium values that depend on the value of . We define these positions , , and , respectively. Equation (5) then becomes

where , , and  are functions, and τ a small constant, which describe the small deviation of the system from the quasi-equilibrium surface. We can left-multiply by the inverse matrix  and concentrate on the evolution of  to give

substituting in for , we can rewrite this expression to first order as

A1

where

We perform a Taylor expansion of  around , , , and , ignoring higher order terms, and get the result , where  for all terms *i*. This gives us

Because we are assuming females choose mates based on *s*, the size of the ornament, genetic correlations , , and  come about through correlations of *t*, , and *v* with *s*. In addition, we have that the genetic correlation between *s* and *p*,  comes about through preference, and is equal to  (Barton and Turelli 1991; Pomiankowski and Iwasa 1993; Tazzyman et al. 2014). So

giving

or, to leading order

so that equation (32) becomes

as given in the main text.

### LARGER ORNAMENTS ARE BETTER SIGNALS

#### Fast dynamics

Equations (7) become

A2

A3

where . We perform a Taylor expansion of ψ around , , , giving

where , , and . Then, assuming we can ignore higher order terms, we get

Because , we can also use a Taylor expansion of  around , ,  to get

where  denotes the first derivative of  with respect to *s*, and , the population average ornament size. We suppose that  is increasing in *s*, but it is locally nearly linear, so that for all *s* we can ignore  or higher derivatives. Then

Equation (40) can then be rewritten as

so from equation (39),

We suppose  and  are small compared to other terms (Barton and Turelli 1991; Iwasa and Pomiankowski 1994), and after some rearrangement we then have, to leading order,

If we suppose that  is of small order relative to *c*_0_, then we get , and a quasi-equilibrium line that can be expressed

A4

where . This is equation (16) in the text. We note that  if and only if . Additionally, we note that

where  is the derivative of  with respect to *t*. If we suppose that  is such that the numerator is always positive (so that  is always increasing in ), then we can say that the quasi-equilibrium line is also expressible as a function of , , and that along this line,

A5

#### Equilibria

Equation (8) becomes

A6

The equilibria are at , and any points where  (eq. 11).

#### **Case**:

At quasi-equilibrium, we have  and . Then equation (8)becomes

By the chain rule,

Thus we can differentiate our expression for  to get

From equation (52) above we have that  evaluated at  is , so evaluating  at  gives , so the equilibrium at the origin is unstable.

#### **Case**:

From equation (18), , and  is strictly decreasing for , and is unbounded above. Because  is strictly increasing, it follows that there is exactly one  such that  (Fig. 1 shows an example). Also, for all , , so from (17), , whereas for , , so  is stable.

#### **Case**:

We suppose for simplicity that there is no continuous subset  so that  for all  (our analysis extends to this case, but for simplicity we omit it here). Then there are two possibilities. Either  for all , so that there are no stable equilibria, and positive runaway occurs. Alternatively, there exists some value  such that , and consequently by continuity there is some , , such that , and  is stable.

### SPECIAL CASE OF VARYING *a* WHEN

Supposing that , we know from Iwasa and Pomiankowski (1994) that

so  when , and also

A7

from equation (52). Using (50) gives us that  when

and this has solutions at  and , defined by

If such a  exists, it must be unique, because  is monotonic and increasing.

Consider the solution . From above we know that this means . Equation (58) then gives that

A8

and from (52) we have that at , . Then equation (61) is negative if the term in brackets on the right-hand side is negative, which is true if

A9

Consequently, if the signaling efficacy *a* [0] of the trait at the naturally selected optimum is below a threshold value , the origin is stable and no sexual signaling will evolve. Because  is monotonically increasing in *t*, we also know that if a solution  exists, then we have  if and only if the origin is stable, and  if and only if the origin is unstable.

Now consider the solution , assuming that it exists. Equations (52) and (58) give

A10

and because we are assuming  for all *t*, (63) has the same sign as . Therefore if , then it is stable, and if , then it is unstable.

Thus, supposing that the system starts at the origin, we have the following two possibilities: (1) Stable origin. There may be an unstable equilibrium point for some . If the system is driven past this point (e.g., by stochastic fluctuations) then it will run away in the direction of exaggeration. Otherwise no signaling will evolve. (2) Unstable origin. The system runs away from the origin in either direction. There may be a stable equilibrium point , in which case evolution toward reduced ornamentation will stop at that point. Evolution toward exaggerated ornamentation will runaway indefinitely. Figure 4 shows the two possibilities.

### PREFERENCE FOR SMALLER ORNAMENTS COSTS MORE

At quasi-equilibrium, (8) becomes

with equilibria given as in the main text. We wish to determine the stability of these equilibria.

#### **Case**:

From above,

so the origin is unstable.

#### **Case**:

From (19), for  such that , , whereas for  such that , , so  is a stable equilibrium.

#### **Case**:

From (19), for  such that , , whereas for  such that , , so  is a stable equilibrium.

### SMALLER ORNAMENTS COST MORE

#### Fast dynamics

Denote by  the derivative of  with respect to the first argument, and by  the derivative with respect to the second argument. Then multiple derivatives will be denoted  for values *i*, *j*, *k*. We denote the derivatives of  with respect to *t*, , and *v* by , , and , respectively. We will again denote multiple derivatives with multiple subscripts. Because , we have

A11

Equations (7) give

A12

A13

Then we can take a Taylor series of  around , , and  to give

where  for all the possible *I* listed. We can then use equation (66) to get

Substituting this, and equation (67) into equation (68), and disregarding terms  as being of much smaller order than , we get that at quasi-equilibrium

A14

Thus, from our definition of  in the text above, we have

Denote , so that . Then, for ,

which gives that on the quasi-equilibrium line, . For , however,

so that . Then because , , and , we have from above, to leading order,

Then we can again use the expression of  from the main text to calculate *c*_111_, *c*_112_, and *c*_122_ as

Then for , we get

Suppose that ε is small, so that we can neglect the  term. Then, to leading order,

because , so . Therefore, at quasi-equilibrium, for ,

For , , and substituting in the expressions for  and , and the quasi-equilibrium equations  and , gives that along the quasi-equilibrium line, .

#### Slow dynamics

For , (8) gives

A15

where

For , (8) gives

A16

The equilibrium points are , , and , where

#### Equilibria

We wish to determine the stability of the equilibria.

#### **Case**:

At , , so the origin is an unstable equilibrium.

#### **Case**:

From equation (83), for  such that , , whereas for  such that , . Therefore,  is a stable equilibrium.

#### **Case**:

From equation (85), for  such that , , whereas for  such that , . Therefore,  is a stable equilibrium.

#### Magnitudes of the equilibria

If  and , then , and because under these circumstances , so , the exaggerated equilibrium will be of greater magnitude than the reduced equilibrium. But for other combinations of parameter values we cannot say anything useful about the magnitudes of the equilibria.

### CONDITION DEPENDENCE

#### *General function*

We take the cost function for a male with viability *v* and ornament size *s* from equation (14), so that *k* is a function of *s*. Then, from equation (71), we know that , which gives us

where  and  are the first and second derivatives of the function . If we suppose that this function is increasing, but very gradually, then we can suppose . The quasi-equilibrium line then satisfies

A17

Unfortunately an explicit solution for this cannot generally be found.

#### Specific function from the main text

We use the function  from equation (15). Then (if we assume  for all *s*, and ignore the discontinuity at ) equation (A17) gives us that at quasi-equilibrium, , and so  for exaggerated ornaments, whereas  for reduced ornaments. Following the above procedures then gives

and because  we have .
